# Far-Infrared Therapy Decreases Orthotopic Allograft Transplantation Vasculopathy

**DOI:** 10.3390/biomedicines10051089

**Published:** 2022-05-07

**Authors:** Yi-Wen Lin, Chien-Sung Tsai, Chun-Yao Huang, Yi-Ting Tsai, Chun-Ming Shih, Shing-Jong Lin, Chi-Yuan Li, Cheng-Yen Lin, Shih-Ying Sung, Feng-Yen Lin

**Affiliations:** 1 Institute of Oral Biology, National Yang Ming Chiao Tung University, Taipei 112, Taiwan; ywlin@nycu.edu.tw; 2 Taipei Heart Institute, Taipei Medical University, Taipei 110, Taiwan; sung1500@mail.ndmctsgh.edu.tw (C.-S.T.); cyhuang@tmu.edu.tw (C.-Y.H.); cmshih53@tmu.edu.tw (C.-M.S.); sjlin@tmu.edu.tw (S.-J.L.); 3 Division of Cardiovascular Surgery, Tri-Service General Hospital, National Defense Medical Center, Taipei 110, Taiwan; cvsallen@mail.ndmctsgh.edu.tw (Y.-T.T.); molecule1983@gmail.com (S.-Y.S.); 4 Department and Graduate Institute of Pharmacology, National Defense Medical Center, Taipei 114, Taiwan; 5 Division of Cardiology and Cardiovascular Research Center, Taipei Medical University Hospital, Taipei 110, Taiwan; 6 Departments of Internal Medicine, College of Medicine, School of Medicine, Taipei Medical University, Taipei 110, Taiwan; 7 Department of Anesthesiology, China Medical University Hospital, Taichung 404, Taiwan; cyli168@gmail.com; 8 Graduate Institute of Clinical Medical Science, China Medical University, Taichung 404, Taiwan; 9 Healthcare Information and Management Department, Ming Chuan University, Taoyuan 333, Taiwan; chengyan@mail.mcu.edu.tw

**Keywords:** orthotopic allograft transplantation, far-infrared (FIR) therapy, endothelial progenitor cells, endothelial mesenchymal transition

## Abstract

Orthotopic allograft transplantation (OAT) is a major strategy for solid heart and kidney failure. However, the recipient’s immunity-induced chronic rejection induces OAT vasculopathy that results in donor organ failure. With the exception of immunosuppressive agents, there are currently no specific means to inhibit the occurrence of OAT vasculopathy. On the other hand, far-infrared (FIR) therapy uses low-power electromagnetic waves given by FIR, with a wavelength of 3–25 μm, to improve human physiological functions. Previous studies have shown that FIR therapy can effectively inhibit inflammation. It has also been widely used in adjuvant therapy for various clinical diseases, especially cardiovascular diseases, in recent years. Thus, we used this study to explore the feasibility of FIR in preventing OAT vasculopathy. In this study, the model of transplantation of an aorta graft from PVG/Seac rat to ACI/NKyo rat, and in vitro model of human endothelial progenitor cells (EPCs) was used. In this report, we presented that FIR therapy decreased the serious of vasculopathy in OAT-recipient ACI/NKyo rats via inhibiting proliferation of smooth muscle cells, accumulation of collagen, and infiltration of fibroblast in the vessel wall; humoral and cell-mediated immune responses were decreased in the spleen. The production of inflammatory proteins/cytokines also decreased in the plasma. Additionally, FIR therapy presented higher mobilization and circulating EPC levels associated with vessel repair in OAT-recipient ACI/NKyo rats. In vitro studies demonstrated that the underlying mechanisms of FIR therapy inhibiting OAT vasculopathy may be associated with the inhibition of the Smad2-Slug axis endothelial mesenchymal transition (EndoMT). Thus, FIR therapy may be the strategy to prevent chronic rejection-induced vasculopathy.

## 1. Introduction

Infrared light is invisible, with a wavelength between 0.75 and 1000 μm, and far-infrared (FIR) is infrared light with a wavelength >3 μm. FIR therapy uses low-power electromagnetic waves with a wavelength of 3–25 μm to improve human physiological functions. Compared to thermal radiation therapy with 0.75–1.5 μm infrared radiation, the extremely low-power electromagnetic waves provided by FIR therapy do not easily cause tissue damage. FIR therapy has been widely used in adjuvant therapy for various clinical diseases in recent years, since an increasing number of studies have shown that it can effectively control the occurrence of inflammation. Results from previous clinical studies suggest that FIR therapy can be used to increase cardiopulmonary exercise tolerance [1,2], improve patency and flow in arteriovenous fistulas in patients with hemodialysis [3] and reduce the probability of re-occlusion within one year after percutaneous transluminal angioplasties [4]. Animal studies have also shown that FIR therapy can increase the expression of heme oxygenase-1 in testes after ischemic injury [5], increase the biological effects of skin microcirculation [6], promote sciatic nerve repair in neuropathy [7], promote ischemia-induced angiogenesis, and restore high glucose-suppressed endothelial progenitor cell (EPC) functions [8]. Previous findings have shown the effectiveness of FIR therapy in the treatment of systemic diseases, including cardiovascular diseases, diabetes mellitus, tissue ischemia, malfunction of native arteriovenous fistulas and prosthetic arteriovenous grafts, chronic pain, and chronic fatigue syndrome [9].

Orthotopic allograft organ transplantation (OAT) have been the major treatment strategy for sever solid organ failure. Given that the presently offered professional approaches can efficiently regulate severe rejection, it has substantially increased the patients’ short-term survival rate after transplanted surgery. Nevertheless, the chronic rejection mainly affects the patient’s long-lasting survival rate.

The alloimmune system attacks the endothelium and epithelium of donor graft, causes diffuse graft damage and results in vasculopathy. This process is called chronic rejection [10]. Arteries, as well as micro vessels, which cause the parenchyma to be replaced by fibrosis, reveal hyperplasia of the vascular intima that raise narrowing and occlusion of the vessels in the transplanted graft [11]. Arterial fibrosis constrains the blood flow, resulting in graft ischemia and failure in patients after organ transplantation [12]. Therefore, OAT vasculopathy results from chronic rejection is a critical issue after organ transplantation.

The immune capacity produced by the systemic immunity is one of the major factors affecting life span of donor graft. The recipient’s immune system recognizes the transplanted organ as an invader, and attempts to exclude it. The appropriate regulation of the immune capacity is crucial for the life span of the transplanted graft, and is also helpful for the recipient. Immunosuppressive therapy is widely used for patients undergoing organ transplantation. Its purpose is to control and regulate damage to the graft by the systemic immune response. Initial immunosuppressive induction and maintenance immunosuppressive therapies are included in immunosuppressive therapy. Initial immunosuppressive induction therapy provides powerful immunosuppressive effects at the hospitalization. Indeed, the success or failure of maintenance immunosuppressive therapy is a major factor in determining patient survival that may regulate chronic rejection. Mycophenolic acids (MPAs), corticosteroids, and calcineurin inhibitors (CNIs) are commonly used drugs for maintenance immunosuppressive therapy [13]. Additionally, mammalian targets of rapamycin inhibitors (mTORi), azathioprine, IL-2 receptor antagonist, monoclonal antibodies, and polyclonal antibodies can also be used in solid organ transplant patients [13,14]. Although multiple drugs are available for the control of immune-related rejection in patients receiving solid organ transplantation, clinicians are still unable to completely and effectively control the progress of chronic rejection and avoid the occurrence of OAT vasculopathy. Therefore, under the current framework of medical treatment, it is necessary to actively search for better preventive strategies for OAT vasculopathy.

FIR therapy is a non-invasive, cheap, and safe procedure that should be easily accepted by patients. Previous studies have shown that FIR therapy has a significant curative effect on inflammatory vascular diseases. Therefore, we aimed to explore the feasibility of FIR therapy for OAT-induced vasculopathy in patients undergoing organ transplantation. In this study, we transplanted the aorta from PVG/Seac rats into the ACI/NKyo rats and compared the severity after 90 days of OAT with and without FIR therapy in animals. We also analyzed the effects of FIR therapy on chronic rejection in animals and investigated the possible mechanisms of FIR therapy on OAT-induced vasculopathy. We hope that the results may increase the suitability of FIR therapy in the adjuvant strategy of prevention in OAT-induced vasculopathy.

## 2. Materials and Methods

### 2.1. Equipment

The FIR therapy device was purchased from WS Far IR Medical Technology Co., Ltd. (Model: TY-101F, Taipei, Taiwan) which provides a far infrared wavelength of 3–25 μm and power intensity of 4.95–20 mW/cm^2^ at a distance of 20 cm. Following the user manual instructions, FIR therapy was administered directly to the backs of the animals or EPCs at a distance of 20 cm.

### 2.2. Animal Study

#### 2.2.1. Authorization of Animal Study

All animals were managed according to the methods accredited by the institutional animal care committee of Taipei Medical University (certification no. LAC-2020-0047). Speculative procedures and animal treatment complied with the “Guide for the Care and Use of Laboratory Animals“ published by the U.S. National Institutes of Health (NIH Publication No. 85–23, revised 1996).

#### 2.2.2. Orthotopic Aortic Transplantation

Since OAT model of PVG/Seac rat-to-ACI/NKyo rat displays vasculopathy that is presented in the previous reference [15,16], therefore this model was used in this study. The 8-week-old and 250–300 g body weight (BW) male ACI/NKyo rats (NBRP rat no. 0001; recipient rats) and PVG/Seac rats (NBRP rat no. 0080; donor rats) were used in this experiment.

#### 2.2.3. Animal Grouping

Total 20 rats were randomly divided into four groups, fed a normal rodent chow diet (scientific diet) and kept in microisolator cages on a 12 h day/night cycle. Group 1 consisted of sham-operated ACI/NKyo rats. Group 2 included OAT-recipient ACI/NKyo rats. Group 3 included OAT-recipient and low-intensity FIR therapy (4.95–8.26 mW/cm^2^) ACI/NKyo rats, and treatment beginning the day after surgery. Group 4 included OAT- recipient and high-intensity FIR therapy (11.7–19.5 mW/cm^2^) ACI/NKyo rats, and treatment beginning the day after surgery. All FIR therapies were performed once daily for 40 min during the experimental period. The transplanted thoracic aortas of rats were removed at day 90 of the experiment.

#### 2.2.4. Biochemical Measurements and Enzyme-Linked Immunosorbent Assays

Plasma levels of creatinine, blood urea nitrogen (BUN), aspartate aminotransferase (AST), alanine transaminase (ALT), lactic dehydrogenase (LDH), and blood sugar were analyzed using a SPOTCHEMTM chemistry system (SP-4410; Arkray, Shanghai, China). C-reactive protein (CRP; Abcam Inc., Cambridge, MA, USA), transforming growth factor β1 (TGF-β1; Abcam Inc., Cambridge, MA, USA), High mobility group box 1 (HMGB1; LifeSpan Biosciences Inc., Seattle, WA, USA), stromal cell-derived factor 1α (SDF-1α; R&D Systems Inc., Minneapolis, MN, USA), interleukin-2 (IL-2; Abcam Inc., Cambridge, MA, USA), and interferon-γ (INF-γ; Abcam Inc., Cambridge, MA, USA) were determined by enzyme-linked immunosorbent assay (ELISA).

#### 2.2.5. Morphological Analysis

After the animals were sacrificed on the 90th experimental day, the transplanted donor aortas spleens were harvested. Tissues were fixed, embedded in paraffin, and cross-sectioned for immunohistochemistry and hematoxylin and eosin (H&E) staining. The spleens were weighed before fixation. Aortas were also stained with Masson’s trichrome and picrosirius red. Immunohistochemical staining of aortas was performed using anti-S100A4 antibody (Cell Signaling Technologies, Danvers, MA, USA) and α-smooth muscle actin antibody (αSMA; Santa Cruz Biotechnology, Dallas, TX, USA) and the spleen was performed using anti-CD138 antibody (Invitrogen, Thermo Fisher Scientific Co., Carlsbad, CA, USA) and anti-CD4, anti-CD8, anti-CD11b, and anti-CD20 antibodies, (Abcam, Cambridge, MA, USA). A light microscope was used to observe the slides.

#### 2.2.6. Flow Cytometry

A flow cytometer was used to analyzed the circulating smooth muscle progenitor cells (SMPCs) and EPCs in rats. Rat blood was incubated with Cy5-conjugated anti-CD34 (Bioss Antibodies, Woburn, MA, USA), Alexa Fluor 488-conjugated anti-CD133 (Novus Biologicals, Centennial, CO, USA), phycoerythrin (PE)-conjugated anti-vascular endothelial growth factor (VEGF; Novus Biologicals, Centennial, CO, USA), and PE-conjugated anti-αSMA (Abcam Inc., Cambridge, MA, USA) antibodies. Isotype IgG was used as a control (Becton Dickinson, Franklin Lakes, NJ, USA). Circulating EPCs were gated using CD133^+^/CD34^+^/VEGF^+^ staining in originate from the monocytic cell. Circulating SMPCs were gated using CD133^+^/αSMA^+^/CD34^−^ staining.

### 2.3. In Vitro Study

#### 2.3.1. Cultivation of Human EPCs and FIR Therapy

Human EPCs were cultured from the total MNCs extracted from the peripheral blood. The protocol was described in a previous report [17]. EPC characterization was performed as previously described. When FIR therapy was required for EPCs, we placed the FIR device in the incubator. We maintained constant humidity and CO_2_ and monitored the temperature with a thermometer (37 °C).

#### 2.3.2. Tubing Formation Assay

In vitro tube formation assays were performed on EPCs to assess the neovasculogenic capacity, which is believed to be important for endothelial function. Human EPCs were treated with recombinant human tumor necrosis factor alpha (TNF-α for 24 h, and FIR therapy was performed simultaneously every 8 h (3 times in 24 h). After the treatment of EPCs was completed, an angiogenesis assay kit (Chemicon, Billerica, MA, USA) was used to investigate the capability of tube formation [18].

#### 2.3.3. Cellular Senescence Assay

Senescence is the negative factor that limits the function of EPCs [19], therefore it was investigated using a cellular senescence assay. The detail protocol was demonstrated in our previous report [20].

#### 2.3.4. Migration Assay (Wound-Healing Assay)

The migration assay was used to study the migratory capacity of EPCs, which is associated with vasculogenesis. The detail protocol was demonstrated in our previous report [20].

#### 2.3.5. Real-Time Quantitative Polymerase Chain Reactions

Quantitative real-time polymerase chain reaction (qPCR) were performed. Glyceraldehyde 3-phosphate dehydrogenase was used as an endogenous control to normalize differences in mRNA expression. The primers are listed in Table 1.

#### 2.3.6. Western Blotting Analysis

The total and nuclear proteins were extracted, and then subjected to sodium dodecyl sulfate-polyacrylamide gel electrophoresis (SDS-PAGE) of Western blotting. Mouse anti-vascular endothelial (VE)-cadherin (Millipore Co., Billerica, MA, USA), rabbit anti-von Willebrand factor (vWF)(Millipore Co., Billerica, MA, USA), mouse anti-αSMA (Sigma-Aldrich, Cambridge, MA, USA), anti-vimentin (Sigma-Aldrich, Cambridge, MA, USA), mouse anti-β-actin (Santa Cruz Biotechnology, Santa Cruz, CA, USA), rabbit anti-phosphorylated Smad2 (Cell Signaling Technology, Danvers, MA, USA), anti-total Smad2/3 (Cell Signaling Technology, Danvers, MA, USA), anti-Snail (Cell Signaling Technology, Danvers, MA, USA), anti-Slug (Cell Signaling Technology, Danvers, MA, USA), and anti-lamin A/C (Cell Signaling Technology, Danvers, MA, USA) antibodies were used. Immunodetection consisted of exposure to an Imaging System of ChemiDoc-ItTM (UVP, Upland, CA, USA).

### 2.4. Statistical Analyses

Values are expressed as mean ± SD. The non-parametric ANOVA, followed by the Kruskal–Wallis test was used to statistical analyses. Results with a *p* < 0.05 were considered statistically significant.

## 3. Results

### 3.1. FIR Therapy Affected the Inflammation-Related Proteins Expression, Not the Biochemical Characteristics, in OAT ACI/NKyo Rats

Biochemical analyses and ELISA were performed to evaluate the effects of FIR therapy in OAT ACI/NKyo rats. The data were showed in Table 2, the body weight, BUN, creatinine, ALT, and AST did not differ between control and experimental groups during the study period. HMGB1 and LDH are associated with antibody-mediated and chronic rejection [21,22]. Significantly, increased the levels of HMGB1 and LDH in OAT ACI/NKyo rats. The data showed that OAT increased LDH (baseline: 750.8 ± 37.3; OAT: 1166.2 ± 111.5 IU/L) and HMGB1 (baseline: 3.2 ± 0.5 ng/mL; OAT: 127.5 ± 89.5 ng/mL) levels. Even with low intensity of FIR therapy, compare to the pre-OAT group (737.6 ± 26.6 IU/L) the level of LDH was still higher (1001.0 ± 142.0 IU/L). In contrast, the increased LDH level was controlled upon therapy with high-intensity FIR in the OAT + FIR group (823.7 ± 57.4 IU/L). Low and high intensity of FIR therapy also decreased the plasma levels of HMGB1 (low intensity of FIR therapy group: 36.5 ± 19.8 ng/mL; high intensity of FIR therapy group: 35.0 ± 10.9 ng/mL) compared to that in the non-FIR therapy group (127.5 ± 89.5 ng/mL) in OAT ACI/NKyo rats. These results indicated that FIR therapy might decrease rejection-mediated tissue damage in OAT ACI/NKyo rats.

### 3.2. FIR Therapy Decreases Vascular Damage and Accumulation of Collagen in OAT ACI/NKyo Rats

**Figure 1:**
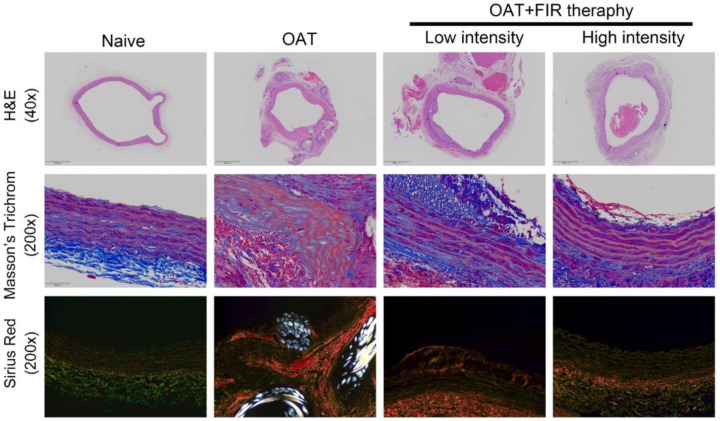
FIR therapy reduced allograft vasculopathy in OAT-recipient ACI/Nkyo rats. (upper column) Thoracic aortas from donor PVG/Seac rats stained with hematoxylin and eosin. The arrows indicate internal elastic lamina and arrowheads indicate calcified lesions. The images are 40× magnified. (middle column) The integrity of collagen fibers of thoracic aorta cross-sections was observed using Masson’s trichome staining. (lower column) Histopathological features and collagen accumulation of thoracic aorta cross-sections were observed using picrosirius red staining. The slides were observed via light microscopy and polarized light microscopy, respectively (200× magnification).

Figure 1 demonstrated the H&E staining of the harvested thoracic aortas. Vasculopathy has actually been linked in the morbid collagen accumulation [23]. Therefore, Masson′s trichrome and picrosirius red staining were used to analyze the collagen phenomena. In fact, no vasculopathy were observed in PVG/Seac rats’ aortas by H&E staining, and intact visualization of the collagen was performed using Masson’s trichrome staining. Additionally, picrosirius red staining showed that thoracic aortas from naïve PVG/Seac rats had thick collagen fibers that presented weak orange to red signal. Additionally, fine collagen fibers were equally distributed (yellow to green) in the vessel walls. Compared to the naïve PVG/Seac rat group, thoracic aortas from recipient OAT ACI/NKyo rats showed vascular integrity damage, blurred elastin laminae, and calcified plaques accumulation after 90 days of OAT. Interestingly, a slightly vascular integrity damage and calcified plaques (vasculopathy) was observed, although slight neointimal formation still existed in OAT ACI/Nkyo rats receiving low-intensity FIR therapy. Furthermore, high-intensity FIR therapy may significantly maintain greater vascular integrity and lower OAT vasculopathy in ACI/Nkyo rats than in non-FIR therapy OAT ACI/Nkyo rats. These results imply that FIR therapy might prevent OAT vasculopathy and promote the integrity of the aortic vessel wall in OAT ACI/Nkyo rats.

### 3.3. Reduced Proliferation of SMCs and Fibroblasts in the Aortic Wall of FIR Therapy-Administered OAT ACI/Nkyo Rats

**Figure 2:**
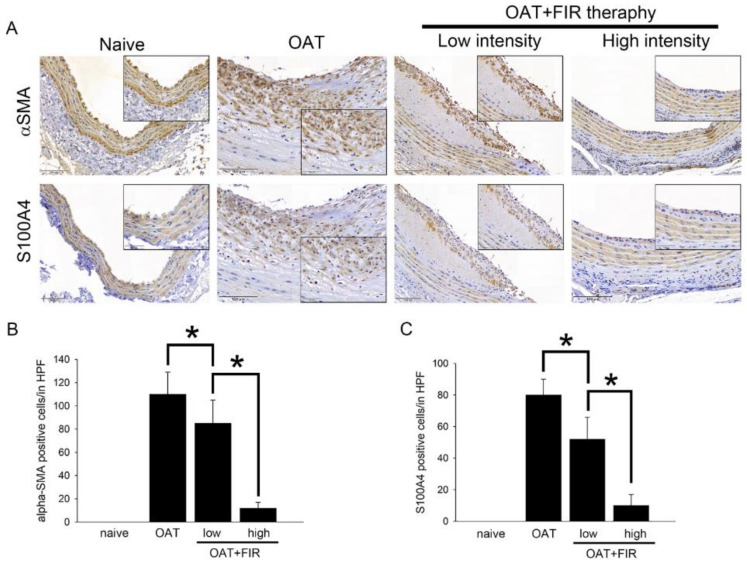
Administration of FIR therapy is effective against SMC and fibroblast activity in OAT-induced chronic allograft vasculopathy. (**A**) Immunohistochemistry to assess proliferated SMCs (αSMA) and fibroblasts (S100A4) in rat thoracic aortas from donor PVG/Seac rats. The lumen is uppermost in all sections; the images are 200× magnified. Similar regions are shown as enlarged images (400× magnification) in the black corners. The brown signal indicates αSMA- and S100A4-positive cells. (**B**,**C**) The quantification of cells in high power field (HPF) is displayed in (**B**,**C**). The graphs demonstrate the accumulation of cells in the aortas of rats. The results are expressed as the mean ± SD. * *p* < 0.05 was taken into consideration statistically considerable.

Smooth muscle cells (SMCs) and fibroblasts proliferation play important roles in allograft vasculopathy. Immunohistochemical staining was used to analyze the effects of FR therapy on SMCs and fibroblast activities. Antibodies against αSMA and S1000A4 on aortic sections were used, and the results are presented in Figure 2. Compared to the PVG/Seac thoracic aorta sections from the naïve group, the sections from the OAT aCI/NKyo rats presented a significant accumulation of fibroblasts and SMCs in the hyperplastic area on the luminal surface at day 90 after transplantation. However, SMCs and fibroblasts accumulated less in the vessel wall in both the low- and high-intensity FIR therapy groups. The efficacy of FIR therapy in inhibiting SMCs and fibroblast proliferation was positively correlated with FIR intensity. These results indicate that minimal inflammation occurred in the aortic wall of the FIR therapy groups, which may have resulted in reduced adaptive immune reaction-related SMCs and fibroblast infiltration.

### 3.4. FIR Therapy Reduced Immune Responses in OAT ACI/NKyo Rats

**Figure 3:**
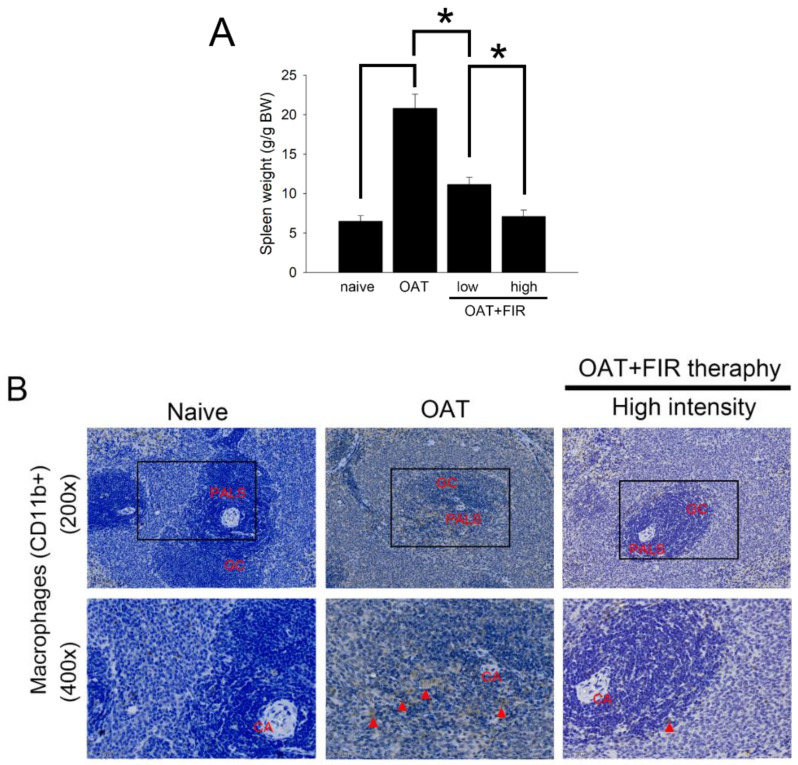

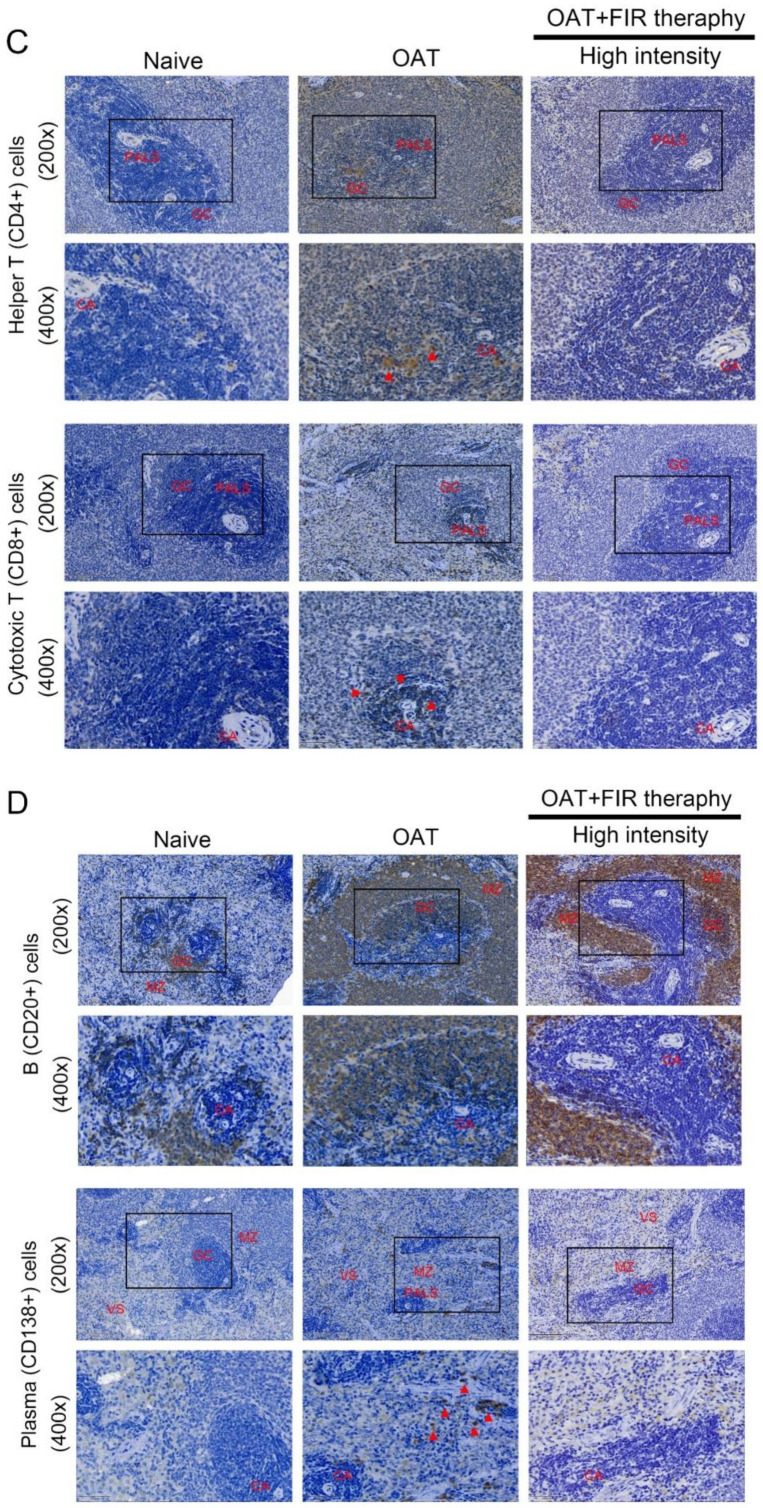
FIR therapy decreased splenic T lymphocytes, plasma cells, B lymphocytes, and macrophages activation in the OAT-ACI/NKyo rats. (**A**) The spleens were dissected from experimental rats after they were sacrificed. The weight of the spleen was analyzed and presented in a bar graph in g/g BW. The results are expressed as the mean ± SD. * *p* < 0.05 was taken into consideration statistically considerable. (**B**) Immunohistochemistry was used to analyze the accumulation of splenic CD11b^+^ macrophages in the OAT-recipient ACI/NKyo rats (CA, central artery; PALS, periarterial lymphatic sheath; GC, germinal center;). The red triangle arrow heads are CD11b^+^ macrophages. The images in the column are 200× and 400× magnification, respectively. (**C**) Immunohistochemistry was used to analyze accumulation of splenic CD8^+^ cytotoxic T cells and CD4^+^ helper T cells in the recipient rats. The images are presented in 200× and 400× magnification. The CD4^+^ and CD8^+^ cells are indicated by red arrow heads. (**D**) The splenic CD20^+^ B cells and CD138^+^ plasma cells accumulation in the OAT-recipient rats (MZ, mantle zone and VS, venous sinuses). The images in the column are 200× and 400× magnification, respectively. The red triangle arrow heads indicate CD138^+^ cells. The cell nuclei were counted with hematoxylin.

Spleen weight was positively correlated with the rejection-related immune response. The spleens of experimental animals were weighed. As shown in Figure 3A, the average weight of the spleen of naive ACI/NKyo rats was 6.5 ± 0.7 g/g BW. The spleens of the OAT ACI/NKyo rats were significantly heavier than those of the naïve ACI/NKyo rats (approximately 20.8 ± 1.8 g/g BW). However, administration of FIR therapy may inhibit the spleen hypertrophy induced by immune response (11.1 ± 0.9 g/g BW in low intensity FIR therapy group and 7.1 ± 0.8 g/g BW in high intensity FIR therapy group). Additionally, the spleens were studied by immunohistochemistry to demonstrate the severity of chronic rejection. CD11b^+^ macrophage is an antigen-presenting cell, that trigger adaptive responses [24]. In Figure 3B, macrophages were merely presented in the splenic periarterial lymphatic sheath (PALS) and germinal center (GC) of naive ACI/NKyo rats. In the non-FIR therapy group after OAT, a lot of macrophages was observed in the PALS and GC. In contrast, OAT with FIR therapy significantly inhibited the macrophages accumulation in splenic GC and PALS. The CD8^+^ killer T cells and CD4^+^ helper T cells regulate cell-mediated immunity. In Figure 3C, the accumulation of helper T cells can be observed in the splenic GC and PALS in naïve ACI/NKyo rats. Additionally, an increased accumulation of helper T cells was presented in the splenic GC and PALS in the OAT without FIR therapy group, in contrast which decreased upon FIR therapy. Similarly, CD8^+^ killer T cells also infiltrated the splenic PALS in the OAT ACI/NKyo rats without FIR therapy, which was twisted by FIR therapy. CD20^+^ B cells regulate humoral immune responses and it can produce antibodies after differentiate into CD138^+^ plasma cells. In Figure 3D, CD20^+^ cells predominantly clustered in the GC and mantle zone and fewer CD138^+^ cells were presented in the splenic GC, venous sinuses, and mantle zone in naïve group. ACI/NKyo rats with only OAT demonstrated with a lot of CD20^+^ cells in the mantle zone and GC, as well as CD138^+^ cells were presented in the splenic venous sinuses. In OAT ACI/NKyo rats, high-intensity FIR therapy resulted in a decreased accumulation of CD20 positive B cells in the GC and mantle zone. Plasma cells were slightly increased, which was observed in the venous sinuses, GC, and mantle zone in OAT ACI/NKyo rats without FIR therapy. FIR therapy may decrease plasma cell accumulation in OAT ACI/NKyo rats. Based on these results, we predicted that FIR therapy might maintain low levels of cell-mediated and humoral immune responses in OAT ACI/NKyo rats.

### 3.5. FIR Therapy Lower Cytokines and Inflammation-Related Proteins Production in OAT ACI/NKyo Rats

The inflammation-related proteins and cytokines in OAT ACI/NKyo rats are shown in Table 3. CRP is an indicator of inflammation and tissue damage [25]; however, the levels were not significantly different between the baseline and 90 days of OAT in all experimental groups. Additionally, EPC function is related to the occurrence of OAT-induced vasculopathy [26]. However, SDF-1α is involved in the homing and recruitment of EPCs, and TNF-α and TGF-β1 negatively regulate EPC function [27] following OAT. Therefore, we also analyzed whether FIR therapy regulates plasma SDF-1α, TNF-α, and TGF-β1 levels, which are associated with the mechanisms of OAT in ACI/NKyo rats (the results are presented in Table 3). OAT induced an increase in SDF-1α in ACI/NKyo rats, with or without FIR therapy, indicating that FIR therapy did not increase SDF-1α production. OAT resulted in a significant TNF-α (243.8 ± 65.2 pg/mL) and TGF-β1 (418.4 ± 102.6 ng/mL) increase at day 90 compared to that of the sham control group (61.6 ± 9.8 pg/mL for TNF-α and 52.5 ± 10.8 ng/mL for TGF-β1). OAT with FIR therapy groups (both low- and high-intensity FIR) also demonstrated lower plasma TNF-α and TGF-β1 levels. Interferon-gamma (IFN-γ) mediates transplant vasculopathy through CD8^+^ or CD4^+^ T lymphocyte-associated injury in vascular endothelial cells. Furthermore, cytokines, such as IL-2, cause a reversible insult to the endothelium at the time of transplantation [28]. Low- and high-intensity FIR therapy may significantly decrease IFN-γ production, and high-intensity FIR therapy may significantly lower interleukin-12 (IL-12) secretion in ACI/NKyo rats after OAT compared to the non-FIR therapy group. Moreover, IFN-γ and IL-12 expression almost reached basal levels in high-intensity FIR therapy OAT ACI/NKyo rats. Based on these results, we predicted that FIR therapy might regulate alloimmunity and nonimmunity factors in appropriate situations.

### 3.6. FIR Therapy Mobilized Circulating EPCs, Not SMPCs, in OAT ACI/NKyo Rats

**Figure 4:**
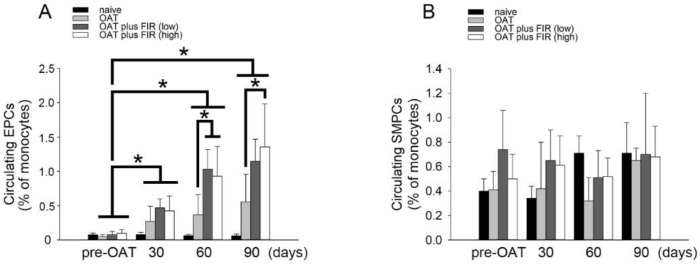
FIR therapy promotes EPCs mobilization in OAT-recipient ACI/NKyo rats. (**A**) CD133^+^/ VEGF^+^/CD34^+^ cells (defined as EPCs) mobilization at day 30–90 following OAT in ACI/NKyo rats were analyzed by flow cytometry. (**B**) CD133^+^/αSMA^+^/CD34^−^ cells (defined as SMPCs) mobilization at day 30–90 following OAT in ACI/NKyo rats were studied. Quantification of EPCs (left) and SMPCs (right) in OAT-recipient rats (black bar, naive rats; light gray bar, OAT only rats; dark gray bar, OAT rats with low intensity of FIR therapy; white bar, OAT rats with high intensity of FIR therapy). All results are expressed as the mean ± SD (*n* = 5). * *p* < 0.05 was taken into consideration statistically considerable.

EPCs play critical roles in the repair of damaged vessels [29]. SDF-1α may trigger the mobilization of circulating EPCs [30]. Additionally, TGF-1β and IFN-γ may command T cell function and may induce endothelial-mesenchymal transition (endo-MT) in the progression of OAT-induced vasculopathy [31,32]. As shown in Table 3, performed the FIR therapy resulted in decreased TGF-1β and INF-γ production in OAT- recipient ACI/NKyo rats. Therefore, following OAT surgery, the population of circulating CD133^+^/CD34^+^/VEGF^+^ EPCs and CD133^+^/αSMA^+^/CD34^−^ SMPCs was analyzed. The results demonstrated significantly increase in EPCs in ACI/NKyo rats compared to naive /non-OAT ACI/NKyo rats on the 30th day after OAT, and it was maintained until on the 90th day after OAT (Figure 4A). On 60th day after OAT, the low- and high-intensity FIR therapy groups increased the EPCs in circulation compared to the non-FIR therapy. However, high-intensity FIR therapy continued to maintain a significantly higher number of circulating EPCs on the 90th day after OAT compared to non-FIR therapy group. Moreover, SMPCs initiate atherosclerosis. However, flow cytometry showed that the circulating SMPCs was not related to FIR therapy (Figure 4B). These results indicate that FIR therapy might promote increased mobilization of early circulating EPCs compared to non-FIR therapy in OAT-recipient ACI/NKyo rats.

### 3.7. FIR Treatment That Regulates the Functions of EPCs May Mediate OAT Vasculopathy

**Figure 5:**
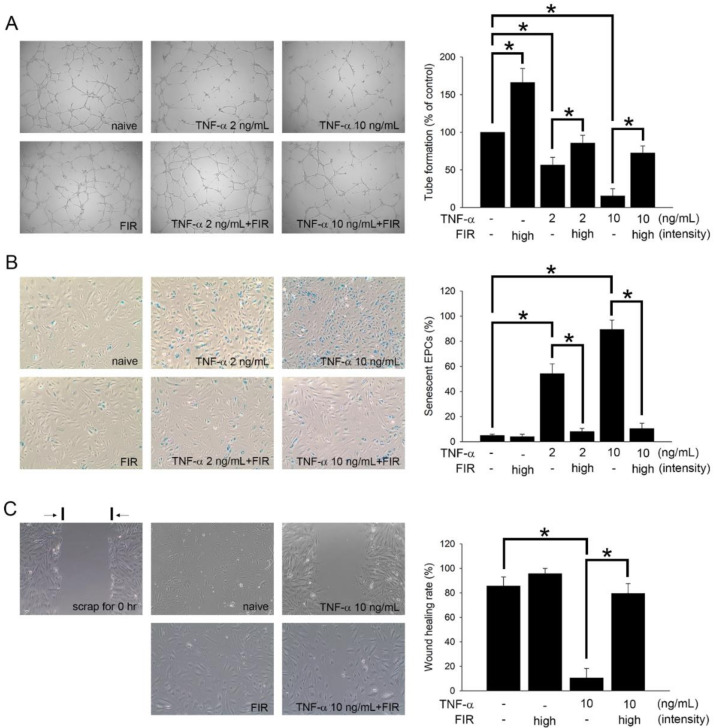
FIR treatment promotes the functions of human EPCs. (**A**) EPCs were stimulated with 2 or 10 ng/mL TNF-α for 24 h with or without high intensity of FIR treatment. An in vitro angiogenesis assay was used to investigate the effect of FIR therapy on EPC neovascularization. Representative photos of in vitro angiogenesis are shown. The graph shows the quantification of tube formation by TNF-α-treated EPCs following FIR treatment. (**B**) After treating EPCs with TNF-α and high intensity of FIR for 24 h, cell senescence was analyzed; the diagram shows the quantification of senescent EPCs. (**C**) A migration assay was performed to analyze the effect of FIR on TNF-α-treated EPCs. The 10 ng/mL of TNF-α were treated to EPCs, and adhered to 24 h of FIR treatment before injury scratching. Photos were taken after 8 h of injuring. Counted the migrated EPCs at the denuded location according to the black baseline under 100× high-power field. All data are expressed as the mean ± SD of three independent experiments and as the percentage of the control. * *p* < 0.05 was taken into consideration statistically considerable.

As the EPCs senescence and function are associated with the OAT vasculopathy [26], we studied the effects of FIR treatment on the activity of EPCs, including tube formation capability, intracellular β-galactosidase activity, and cellular migratory performance. As shown in Figure 4A and Table 3, FIR therapy increased the differentiation of mononuclear cells into circulating EPCs in ACI/NKyo rats, and also decreased the plasma level of the cytokine TNF-α in OAT ACI/NKyo. We hypothesized that FIR therapy decreases plasma TNF-α levels and is associated with increased EPC function and activity. Figure 5 shows the results of the in vitro study. After 24 h of treatment with 2 or 10 ng/mL TNF-α, the tube-forming phenomena of EPCs was significantly decreased compared to that of the control (2 ng/mL TNF-α group: 56.4 ± 10.2% of the control; 10 ng/mL TNF-α group: 15.4 ± 9.7% of the control). In contrast, in the group of FIR treatment, the tube-forming phenomena was significantly increased (2 ng/mL TNF-α with FIR treatment group: 85.7 ± 10.3% of the control; 10 ng/mL TNF-α with FIR treatment group: 72.4 ± 9.4% of the control) compared with that of the 2 or 10 ng/mL TNF-α groups (Figure 5A). Additionally, Figure 5B shows that compared to the control group, senescence increased following TNF-α treatment (2 ng/mL TNF-α group: 54.2 ± 7.8% of the control; 10 ng/mL TNF-α group: 89.4 ± 7.5% of the control). However, compared to the FIR-treated groups, FIR treatment significantly inhibited the presentation of β-galactosidase-positive EPCs under TNF-α stimulation (2 ng/mL TNF-α with FIR treatment group: 8.1 ± 2.5% of the control; 10 ng/mL TNF-α with FIR treatment group: 10.5 ± 4.2% of the control). In addition, a migration assay was performed to study the effect of FIR treatment on TNF-α-treated EPCs. The EPCs were then cultured in the presence of TNF-α and FIR treatment, and images were taken 8 h after wounding. Significantly, 10 ng/mL TNF-α decreased the wound closure rate (10.5 ± 7.8%) compared to that in the control group (85.6 ± 7.4%), whereas FIR treatment significantly reversed the decline (79.5 ± 8.1%) (Figure 5C). FIR treatment increased the tube formation capability of naive EPCs but had no significant effect on β-galactosidase activity and migration activity of naive EPCs. These results indicate that FIR treatment might effectively promote these functions and prevented EPC senescence.

### 3.8. FIR Treatment Regulates the EndoMT of EPCs and May Mediate OAT Vasculopathy

**Figure 6:**
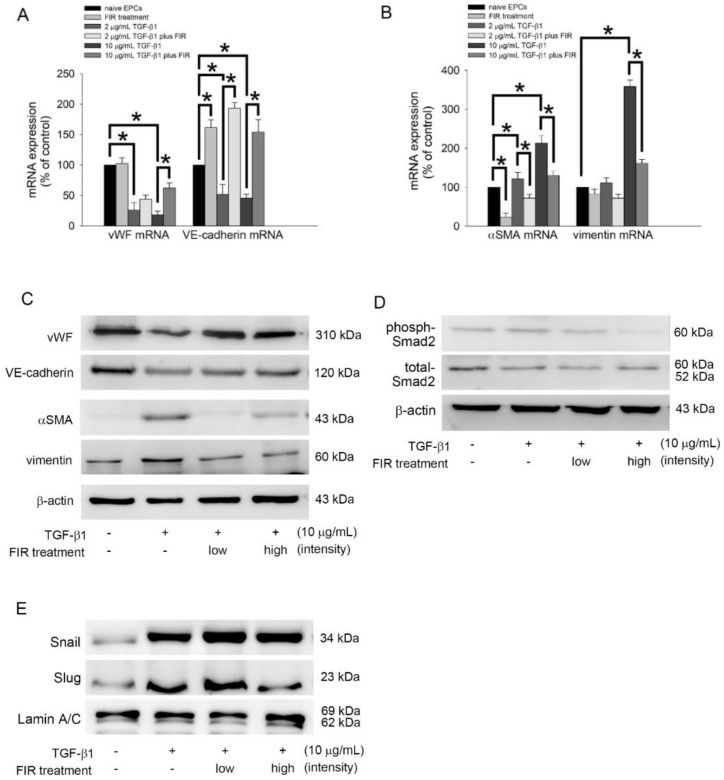
FIR treatment regulates TGF-β1-induced EndoMT via Smad- and Slug-dependent pathways. (**A**,**B**) Human EPCs were exposed to 2 or 10 μg/mL recombinant human TGF-β1 for 5 days with high intensity or without FIR treatment. The α-SMA, VE-cadherin, vWF, and vimentin mRNA expression were evaluated using reverse transcription and qPCR analysis. The expression of related mRNA expression is normalized to the expression of GAPDH mRNA, is presented as a bar graph. All data are expressed as the mean ± SD of five independent experiments and as the percentage of the control. * *p* < 0.05 was taken into consideration statistically considerable. (**C**,**D**) Human EPCs were exposed to 10 μg/mL TGF-β1 for 5 days with low intensity, high intensity or without FIR treatment. The total protein expression of the vWF, VE-cadherin, α-SMA, vimentin, and phosphorylated Smad2 were identified by Western blot analysis. β-actin and total-Smad2 were used as loading controls. (**E**) Human EPCs were treated with 10 μg/mL TGF-β1 in the presence or absence of FIR treatment for 5 days. Total nuclear lysates were purified, and the levels of Snail and Slug were analyzed using Western blotting; lamin A/C was used as a loading control.

As the endothelial to mesenchymal transition (EndoMT) of EPCs is associated with the process of OAT vasculopathy [33], Table 3 presents that OAT increased the plasma TGF-β1 level and reversed by FIR therapy in ACI/NKyo rats. Therefore, we hypothesized that FIR therapy may decrease plasma TGF-β1-induced EndoMT associated with OAT. We investigated the effects of FIR treatment on the EndoMT of EPCs, including the expression of related factors (vWF, VE-cadherin, αSMA, and vimentin). As shown in Figure 6A, treatment with 2 or 10 μg/mL TGF-β1 for 5 days decreased the expression of vWF mRNA and VE-cadherin mRNA compared to the control (naive group). Therapy with high-intensity FIR could reverse the decline in expression of vWF mRNA and VE-cadherin mRNA in TGF-β1 culture. In contrast, TGF-β1 significantly increased αSMA mRNA and vimentin mRNA expression, which was significantly prevented by FIR treatment (Figure 6B). Additionally, Western blot analysis demonstrated that FIR treatment increased vWF and VE-cadherin expression but reversed αSMA and vimentin expression in TGF-β1-treated EPCs (Figure 6C). Smad2 phosphorylation is associated with EndoMT. Therefore, Western blotting was performed to explore the effects of FIR therapy on Smad2 expression in TGF-β1-induced EPCs. Figure 6D presented that high-intensity FIR therapy decreased Smad2 phosphorylation in TGF-β1-treated EPCs. Transcription factors, such as Snail and Slug, positively regulate the markers expression of EndoMT [34] and mediate the loss of cellular adhesion in endothelial cells [35]. Therefore, we investigated the effect of FIR treatment on the activation of Snail and Slug. In Figure 6E, TGF-β1 increased the activation of Snail and Slug, and FIR treatment inhibited the nuclear translocation of Slug. However, FIR treatment did not affect the activation of Snail in TGF-β1-stimulated EPCs. According to these results, we conclude that high-intensity FIR can effectively and stably inhibit EndoMT by controlling the phosphorylation of Smad2 and activation of Slug transcription factors in EPCs; however, the role of other signaling pathways that were not analyzed in this study cannot be neglected.

## 4. Discussion

There are many ways to implement FIR therapy in clinical practice [6,36,37], and they always provide thermal and non-thermal effects to increase blood flow [38,39], maintain endothelial function, lower blood pressure [40,41], and regulate nerve function [42,43]. Compared to the other diseases, FIR therapy, has a significant impact on cardiovascular diseases. Ikeda et al. showed that FIR therapy can increase endothelial nitric oxide synthase (eNOS) mRNA expression, eNOS protein production, and nitric oxide (NO) levels in cardiomyopathy and heart failure [44], which may be related to the pathway of increasing Ca^2+^/calmodulin-dependent protein kinase (CaMKII)-mediated eNOS phosphorylation [45]. Increasing eNOS activity and NO content can effectively improve vascular endothelial and cardiac function, increase cardiopulmonary exercise tolerance [1,2], and inhibit platelet aggregation and smooth muscle cell migration/proliferation [46]. Additionally, FIR therapy reduces plasma levels of lipid peroxidation and 8-epi-prostaglandin F_2__α_ [47]. The 8-epi-prostaglandin F_2__α_ causes systemic oxidative stress and subsequently induces atherosclerosis and congenital heart failure. NO production can be increased by reducing 8-epi-prostaglandin F_2__α_ levels and its oxidative stress [48]. This may also be the mechanism by which FIR therapy improves vascular endothelial cell function and prevents the occurrence of cardiovascular diseases. EPC differentiation and mobilization in OAT rats is one of the factors that determines vasculopathy [26]. Additionally, eNOS activity and NO production can modify the differentiation and mobilization of EPCs [49]. In the present study, we found that FIR therapy increased the functions of EPCs, including migration and tube formation capacity, in OAT rats. Although we did not currently analyze the effect of FIR therapy on NO activity in rats after OAT, based on the results of a previous study conducted by our group which found that FIR therapy reduced oxidative stress and upregulated NO bioavailability in streptozotocine-induced diabetic mice [8], we speculate that the vasculopathy prevented by FIR therapy may be related to the regulation of NO activity.

In this experiment, we performed FIR therapy with an FIR emitter, consisting of electrified ceramic plates, and irradiated 20 cm from the skin for 40 min for each cycle. FIR therapy provides low energy to steadily increase the skin temperature. We cannot rule out thermal effects and an effect on the occurrence of OATV in animals following increased skin temperature. However, we controlled the temperature of the incubator and experimented with cells at 37 °C in an in vitro study. Therefore, we can speculate that the effects of FIR therapy on cells and tissues are due to its nonthermal effects. Previous reports have demonstrated that miRNAs are involved in the development of the cardiovascular system [50] and regulate the occurrence of cardiovascular diseases and function of vascular endothelial cells [50,51,52]. Plasma miRNAs, such as miRNA-1, miRNA-17, miRNA-21, miRNA-92a, miRNA-126, miRNA-133, and miRNA-145, have been considered as markers of cardiovascular diseases [51,53] and indicators to estimate the course of acute myocardial infarction [54]. In addition, the functions of EPCs, including proliferation, migration, senescence, apoptosis, mobilization, and differentiation, are regulated by many miRNAs [55]. Therefore, we speculate that FIR therapy may modulate the function of EPCs by altering the expression of miRNAs under pathological conditions to avoid vasculopathy in patients with OAT. We are analyzing the possible effects of FIR on the expression of miRNAs in EPC, and thus to understand the possible roles of miRNAs in the process of FIR treatment of OAT.

The incident of chronic rejection after OAT refer to the manufacturing of anti-bodies versus donor-specific leukocyte antigens (HLA) by the recipient [56]. Donor antigen-presenting cells existing in the tissue, such as the MHC fragments on the surface of dendritic cells, will absolutely be recognized by the T cells in the recipient, which subsequently induces the cellular immune response. On top of that, antigen fragments from donor provided externally to the recipient′s antigen-presenting cells are acknowledged by the T cells in recipient [12]. This procedure is the major initiator of the immune response for chronic rejection [57]. The release of IFN-γ from activated T cells will continue to activate B cells and macrophages, and additionally amplify the endothelial cells in the graft to express cellular adhesion molecules. SMCs are also proliferation [58] and secret extracellular matrix proteins resulting from simultaneous activation. Concomitantly, anti-HLA antibodies are produced by activated B cells, which promote vasculopathy in the donor graft. In this study, we clearly observed that the concentration of IFN-γ in the plasma of OAT rats treated with FIR therapy was significantly reduced, and the infiltration of SMCs in the donor aorta was significantly inhibited compared with that in the group without FIR therapy. Immunohistochemical staining also showed that FIR therapy reduced T and B cell activity in the spleen. We are the first group to publish on FIR therapy for the suppression of chronic rejection after OAT. In addition to the clinical use of immunosuppressive and immunomodulatory agents to control autoimmune diseases or chronic rejection, the adjunctive use of FIR therapy may be a way to make traditional treatments more effective.

EPCs play an important role associated with the process of OAT vasculopathy [59]. Endo-MT, which describes the procedure where ECs differentiate into fibroblasts and also SMCs [60,61]. TGF-β1 regulates the development of fibrosis [61,62]. EPCs advertise healing and also repair of harmed endothelium [63] also keep vascular endothelial function [64]. However, current researchers have actually discovered that EPCs in the patient underwent heart transplantation are associated with the formation of vasculopathy [57,64,65]. EPCs from the recipient adhere to the vessel wall of the transplanted organ and begin EndoMT, leading to alloimmune responses following OAT [65]. Alloimmune responses can result in serious EPCs EndoMT and subsequent accumulation of SMCs and fibroblasts [65,66], leading to an excessive accumulation of extracellular matrix and neointimal formation [64]. Our research results show that FIR therapy can reduce the levels of cytokines in the plasma that induce cell-mediated and humoral immune responses, such as IL-12 and INF-γ, and reduce the attack on donor grafts after T cell and B cell activation in OAT ACI/NKyo rats. The incidence of OAT vasculopathy was reduced by reducing TGF-β-induced EndoMT via the Smad2-Slug-axis signaling pathway. Based on these results, we believe that FIR therapy might have the potential to be used more widely in the treatment of diseases related to immune system abnormalities or impaired EPCs function.

## 5. Conclusions

**Figure 7:**
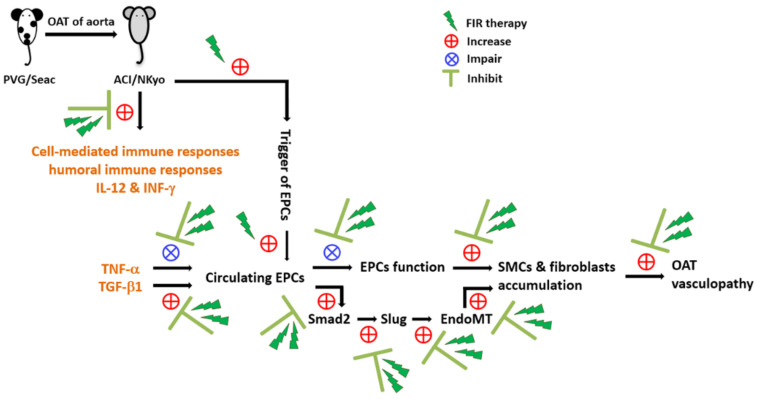
FIR therapy may effectively regulate chronic rejection-induced vasculopathy in OAT rats. Therapy of FIR reduced T and B lymphocytes, plasma cells, and macrophage activation in the spleens of the OAT-recipient ACI/NKyo rats. Lowered the progression of vasculopathy in OAT-recipient ACI/NKyo rats occurred by the inhibition of cell-mediated and humoral immune responses, prevention of cytokines-induced disfunction and EndoMT in EPCs, decrease in collagen damage and pathological accumulation, and proliferation and infiltration of SMCs and fibroblasts in the vessel wall of OAT-recipient ACI/NKyo rats. Therefore, the results highlight the therapeutic roles of FIR and provides a more effective adjuvant therapeutic route in vasculopathy.

We conclude the results of this study with a scheme diagram (Figure 7). The animal study of OAT-induced vasculopathy in ACI/NKyo rats revealed that FIR therapy could prevent vasculopathy via anti-immune responses and anti-inflammatory mechanisms. In contrast, FIR therapy could increase the number of circulating EPCs in OAT ACI/NKyo rats. In vitro experiments have also confirmed that FIR can reduce the negative effects (such as EndoMT and senescence) of cytokines (such as TNF-α, TGF-β1, and INF-γ) on EPCs and increase their activity. These mechanisms are associated with the occurrence of OAT vasculopathy. Thus, this study might provide new insights into the preventive strategy of using FIR therapy to treat chronic rejection-induced vasculopathy.

## Figures and Tables

**Table 1:** The primer sequence for real-time RCR.

Gene	Forward Primer	Reverse Primer
vWF	5′-GGC TGC AGT ATG TCA AGG TGG-3′	5′-AGA GCC ATT GGT GCA GTG CAG-3′
VE-cadherin	5′-AGA CAA TGG GAT GCC AAG TCB-3′	5′-AAG ATG AGC AGG GTG ATC ACT G-3′
αSMA	5′-CTA TCA GGG GGC ACC ACT ATG-3′	5′-CCG ATC CAG ACA GAG TAT TTG CG-3′
vimentin	5′-AGG CAA AGC AGG AGT CCA CTG A-3′	5′-ATC TGG CGT TCC AGG GAC TCAT -3′
GAPDH	5′-TGC CCC CTC TGC TGA TGC C-3′	5′-CCT CCG ACG CCT GCT TCA CCA C-3′

vWF, von Willebrand factor; αSMA, alpha smooth muscle cell actin; GAPDH, glyceraldehyde 3-phosphate dehydrogenase.

**Table 2:** Comparison of biochemical parameters in experimental ACI/NKyo rats (*n* = 5).

	Sham Control		OAT		OAT+FIR Low Intensity		OAT+FIR High Intensity	
	baseline	90 days	baseline	90 days	baseline	90 days	baseline	90 days
Body weight (g)	256.8 ± 12.8	340.2 ± 9.5	270.8 ± 10.8	341.6 ± 10.7	258.4 ± 7.6	345.8 ± 4.4	266.2 ± 16.4	346.0 ± 13.5
BUN (mg/dL)	26.5 ± 1.4	29.6 ± 2.4	30.4 ± 2.4	29.6 ± 1.7	28.7 ± 3.1	29.7 ± 2.3	30.5 ± 2.9	33.7 ± 2.3
Creatinine (mg/dL)	0.4 ± 0.1	0.6 ± 0.1	0.6 ± 0.1	0.5 ± 0.2	0.5 ± 0.1	0.6 ± 0.1	0.6 ± 0.2	0.4 ± 0.2
ALT (IU/L)	26.3 ± 1.6	26.0 ± 1.8	27.7 ± 1.8	28.8 ± 1.2	26.7 ± 1.1	28.0 ± 1.6	26.2 ± 0.9	26.4 ± 1.8
AST (IU/L)	34.4 ± 1.3	33.8 ± 1.3	34.4 ± 1.8	34.6 ± 1.4	33.9 ± 1.2	32.6 ± 1.7	35.2 ± 2.5	34.5 ± 1.7
LDH (IU/L)	786.6 ± 37.8	732.7 ± 20.3	750.8 ± 37.3	1166.2 ± 111.5 ^ab^	737.6 ± 26.6	1001.0 ± 142.0 ^ab^	757.1 ± 30.5	823.7 ± 57.4 ^c^
HMGB1 (ng/mL)	3.3 ± 0.7	3.4 ± 0.5	3.2 ± 0.5	127.5 ± 89.5 ^ab^	2.4 ± 0.9	36.5 ± 19.8 ^abc^	2.5 ± 0.5	35.0 ± 10.9 ^abc^

FIR, far-infrared ray; BW, body weight; OAT, orthotopic aortic transplantation; BUN, blood urea nitrogen; ALT, alanine transaminase; AST, aspartate transaminase; LDH, lactic dehydrompared. HMGB1, high mobility group box 1 protein. ^a^ *p* < 0.05 compared with baseline of the same group; ^b^ *p* < 0.05 compared with sham control ACI/NKyo (non-OAT) group at the same time point; ^c^ *p* < 0.05 compared with OAT (PVG/Seac to ACI/NKyo) group at the same time point.

**Table 3:** Comparison of OAT vasculopathy-related factors in ACI/NKyo rats (*n* = 5).

	Sham Control		OAT		OAT+FIR Low Intensity		OAT+FIR High Intensity	
Proteins	baseline	90 days	baseline	90 days	baseline	90 days	baseline	90 days
CRP (mg/dL)	30.9 ± 6.1	33.5 ± 14.6	33.6 ± 12.5	39.4 ± 8.5	32.6 ± 6.7	32.0 ± 11.7	33.9 ± 13.1	27.9 ± 10.5
SDF-1α (pg/mL)	180.5 ± 42.4	192.2 ± 41.1	200.7 ± 50.8	376.8 ± 103.2 ^ab^	169.1 ± 63.9	367.2 ± 81.5 ^ab^	259.3 ± 95.5	444.2 ± 106.9 ^ab^
TNF-α (pg/mL)	68.2 ± 11.4	61.6 ± 9.8	59.0 ± 9.3	243.8 ± 65.2 ^ab^	62.0 ± 7.0	110.9 ± 44.2 ^abc^	70.1 ± 10.8	113.2 ± 47.7 ^c^
TGF-β1 (ng/mL)	50.6 ± 11.2	52.5 ± 10.8	46.6 ± 13.3	418.4 ± 102.6 ^ab^	41.6 ± 16.9	155.1 ± 54.9 ^abc^	39.0 ± 7.3	136.3 ± 54.6 ^abc^
INF-γ (pg/mL)	2.9 ± 0.8	2.9 ± 0.7	3.5 ± 1.0	23.2 ± 7.5 ^ab^	3.5 ± 1.3	15.5 ± 3.8 ^abc^	3.3 ± 1.5	4.7 ± 1.4 ^bc^
IL-12 (pg/mL)	195.5 ± 38.9	181.6 ± 32.1	202.5 ± 34.7	404.6 ± 88.7 ^ab^	176.7 ± 58.8	327.6 ± 60.3 ^ab^	191.3 ± 45.4	187.7 ± 49.9 ^c^

FIR, far-infrared ray; OAT, orthotopic aortic transplantation; CRP, C-reactive protein; TNF-α, tumor necrosis factor-alpha; SDF-1α, stromal cell-derived factor 1 alpha; INF-γ, interferon gama; IL-12, interleukin 12; TGF-β1, transforming growth factor- beta 1; Values are represented as mean ± SD. ^a^ *p* < 0.05 compared with baseline of the same group; ^b^ *p* < 0.05 compared with sham control ACI/NKyo (non-OAT) group at the same time point; ^c^ *p* < 0.05 compared with OAT (PVG/Seac to ACI/NKyo) group at the same time point.

## Data Availability

The data presented in this study are available on request from the corresponding author.

## References

[1] Sobajima M., Nozawa T., Ihori H., Shida T., Ohori T., Suzuki T., Matsuki A., Yasumura S., Inoue H. (2013). Repeated sauna therapy improves myocardial perfusion in patients with chronically occluded coronary artery-related ische-mia. Int. J. Cardiol..

[2] Ohori T., Nozawa T., Ihori H., Shida T., Sobajima M., Matsuki A., Yasumura S., Inoue H. (2012). Effect of repeated sauna treatment on exercise tolerance and endothelial function in patients with chronic heart failure. Am. J. Cardiol..

[3] Lin C.C., Chang C.F., Lai M.Y., Chen T.W., Lee P.C., Yang W.C. (2007). Far-infrared therapy: A novel treatment to im-prove access blood flow and unassisted patency of arteriovenous fistula in hemodialysis patients. J. Am. Soc. Nephrol..

[4] Lai C.C., Fang H.C., Mar G.Y., Liou J.C., Tseng C.J., Liu C.P. (2013). Post-angioplasty far infrared radiation therapy improves 1-year angioplasty-free hemodialysis access patency of recurrent obstructive lesions. Eur. J. Vasc. Endovasc. Surg..

[5] Tu Y.P., Chen S.C., Liu Y.H., Chen C.F., Hour T.C. (2013). Postconditioning with far-infrared irradiation increases heme oxygenase-1 expression and protects against ischemia/reperfusion injury in rat testis. Life Sci..

[6] Yu S.Y., Chiu J.H., Yang S.D., Hsu Y.C., Lui W.Y., Wu C.W. (2006). Biological effect of far-infrared therapy on increas-ing skin microcirculation in rats. Photodermatol. Photoimmunol. Photomed..

[7] Chen T.Y., Yang Y.C., Sha Y.N., Chou J.R., Liu B.S. (2015). Far-Infrared Therapy Promotes Nerve Repair following End-to-End Neurorrhaphy in Rat Models of Sciatic Nerve Injury. Evid. Based Complement. Altern. Med..

[8] Huang P.H., Chen J.W., Lin C.P., Chen Y.H., Wang C.H., Leu H.B., Lin S.J. (2012). Far infra-red therapy promotes is-chemia-induced angiogenesis in diabetic mice and restores high glucose-suppressed endothelial progenitor cell functions. Cardiovasc. Diabetol..

[9] Shui S., Wang X., Chiang J.Y., Zheng L. (2015). Far-infrared therapy for cardiovascular, autoimmune, and other chronic health problems: A systematic review. Exp. Biol. Med..

[10] Lund L.H., Edwards L.B., Kucheryavaya A.Y., Dipchand A.I., Benden C., Christie J.D., Dobbels F., Kirk R., Rahmel A.O., Yusen R.D. (2013). The Registry of the International Society for Heart and Lung Transplantation: Thir-tieth Official Adult Heart Transplant Report--2013; focus theme: Age. J. Heart Lung Transplant..

[11] Costello J.P., Mohanakumar T., Nath D.S. (2013). Mechanisms of chronic cardiac allograft rejection. Tex. Heart Inst. J..

[12] Nath D.S., Basha H.I., Mohanakumar T. (2010). Antihuman leukocyte antigen antibody-induced autoimmunity: Role in chronic rejection. Curr. Opin. Organ Transplant..

[13] Holt C.D. (2017). Overview of Immunosuppressive Therapy in Solid Organ Transplantation. Anesthesiol. Clin..

[14] Jasiak N.M., Park J.M. (2016). Immunosuppression in Solid-Organ Transplantation: Essentials and Practical Tips. Crit. Care Nurs. Q..

[15] Poston R.S., Billingham M., Hoyt E.G., Pollard J., Shorthouse R., Morris R.E., Robbins R.C. (1999). Rapamycin reverses chronic graft vascular disease in a novel cardiac allograft model. Circulation.

[16] Bedi D.S., Riella L.V., Tullius S.G., Chandraker A. (2010). Animal models of chronic allograft injury: Contributions and limitations to understanding the mechanism of long-term graft dysfunction. Transplantation.

[17] Chen Y.H., Lin S.J., Lin F.Y., Wu T.C., Tsao C.R., Huang P.H., Liu P.L., Chen Y.L., Chen J.W. (2007). High glucose impairs early and late endothelial progenitor cells by modifying nitric oxide-related but not oxidative stress-mediated mechanisms. Diabetes.

[18] Chen J.Z., Zhu J.H., Wang X.X., Xie X.D., Sun J., Shang Y.P., Guo X.G., Dai H.M., Hu S.J. (2004). Effects of homocys-teine on number and activity of endothelial progenitor cells from peripheral blood. J. Mol. Cell. Cardiol..

[19] Goldstein S. (1990). Replicative senescence: The human fibroblast comes of age. Science.

[20] Lin F.Y., Shih C.M., Huang C.Y., Tsai Y.T., Loh S.H., Li C.Y., Lin C.Y., Lin Y.W., Tsai C.S. (2021). Dipeptidyl Pepti-dase-4 Inhibitor Decreases Allograft Vasculopathy Via Regulating the Functions of Endothelial Progenitor Cells in Normoglycemic Rats. Cardiovasc. Drugs Ther..

[21] Zou H., Yang Y., Gao M., Zhang B., Ming B., Sun Y., Chen H., Tang X., Chen Z., Xiong P. (2014). HMGB1 is involved in chronic rejection of cardiac allograft via promoting inflammatory-like mDCs. Am. J. Transplant..

[22] Khan T.T., Mirza A.B., Zahid R., Haleem A., Al Hussaini H., Al Sulaiman M., Mousa D. (2011). Antibody-mediated rejection: Importance of lactate dehydrogenase and neutrophilia in early diagnosis. Saudi J. Kidney Dis. Transplant..

[23] Kato G.J., Hebbel R.P., Steinberg M.H., Gladwin M.T. (2009). Vasculopathy in sickle cell disease: Biology, pathophysi-ology, genetics, translational medicine, and new research directions. Am. J. Hematol..

[24] Rua R., McGavern D.B. (2015). Elucidation of monocyte/macrophage dynamics and function by intravital imaging. J. Leukoc. Biol..

[25] Sproston N.R., Ashworth J.J. (2018). Role of C-Reactive Protein at Sites of Inflammation and Infection. Front. Immunol..

[26] Fadini G.P., Sartore S., Albiero M., Baesso I., Murphy E., Menegolo M., Grego F., Vigili de Kreutzenberg S., Tiengo A., Agostini C. (2006). Number and function of endothelial progenitor cells as a marker of severity for dia-betic vasculopathy. Arterioscler. Thromb. Vasc. Biol..

[27] Pintavorn P., Ballermann B.J. (1997). TGF-beta and the endothelium during immune injury. Kidney Int..

[28] Weis M., Wildhirt S.M., Schulze C., Pehlivanli S., Fraunberger P., Meiser B.M., von Scheidt W. (1999). Modulation of coronary vasomotor tone by cytokines in cardiac transplant recipients. Transplantation.

[29] Del Papa N., Pignataro F. (2018). The Role of Endothelial Progenitors in the Repair of Vascular Damage in Systemic Sclerosis. Front. Immunol..

[30] Tilling L., Chowienczyk P., Clapp B. (2009). Progenitors in motion: Mechanisms of mobilization of endothelial progen-itor cells. Br. J. Clin. Pharmacol..

[31] Oh S.A., Li M.O. (2013). TGF-beta: Guardian of T cell function. J. Immunol..

[32] Knight R.J., Liu H., Fishman E., Reis E.D. (2003). Cold ischemic injury, aortic allograft vasculopathy, and pro-inflammatory cytokine expression. J. Surg. Res..

[33] Kovacic J.C., Dimmeler S., Harvey R.P., Finkel T., Aikawa E., Krenning G., Baker A.H. (2019). Endothelial to Mesen-chymal Transition in Cardiovascular Disease: JACC State-of-the-Art Review. J. Am. Coll. Cardiol..

[34] Mahmoud M.M., Serbanovic-Canic J., Feng S., Souilhol C., Xing R., Hsiao S., Mammoto A., Chen J., Ariaans M., Francis S.E. (2017). Shear stress induces endothelial-to-mesenchymal transition via the transcription factor Snail. Sci. Rep..

[35] Platel V., Faure S., Corre I., Clere N. (2019). Endothelial-to-Mesenchymal Transition (EndoMT): Roles in Tumorigene-sis, Metastatic Extravasation and Therapy Resistance. J. Oncol..

[36] Tei C. (2007). Waon therapy: Soothing warmth therapy. J. Cardiol..

[37] Tei C., Horikiri Y., Park J.C., Jeong J.W., Chang K.S., Toyama Y., Tanaka N. (1995). Acute hemodynamic improve-ment by thermal vasodilation in congestive heart failure. Circulation.

[38] Akasaki Y., Miyata M., Eto H., Shirasawa T., Hamada N., Ikeda Y., Biro S., Otsuji Y., Tei C. (2006). Repeated thermal therapy up-regulates endothelial nitric oxide synthase and augments angiogenesis in a mouse model of hindlimb ischemia. Circ. J..

[39] Ise N., Katsuura T., Kikuchi Y., Miwa E. (1987). Effect of far-infrared radiation on forearm skin blood flow. Ann. Physiol. Anthropol..

[40] Kihara T., Biro S., Imamura M., Yoshifuku S., Takasaki K., Ikeda Y., Otuji Y., Minagoe S., Toyama Y., Tei C. (2002). Repeated sauna treatment improves vascular endothelial and cardiac function in patients with chronic heart failure. J. Am. Coll. Cardiol..

[41] Ryotokuji K., Ishimaru K., Kihara K., Namiki Y., Hozumi N. (2013). Effect of pinpoint plantar long-wavelength infra-red light irradiation on subcutaneous temperature and stress markers. Laser Ther..

[42] Su L.H., Wu K.D., Lee L.S., Wang H., Liu C.F. (2009). Effects of far infrared acupoint stimulation on autonomic activ-ity and quality of life in hemodialysis patients. Am. J. Chin. Med..

[43] Oosterveld F.G., Rasker J.J., Floors M., Landkroon R., van Rennes B., Zwijnenberg J., van de Laar M.A., Koel G.J. (2009). Infrared sauna in patients with rheumatoid arthritis and ankylosing spondylitis. A pilot study showing good tolerance, short-term improvement of pain and stiffness, and a trend towards long-term beneficial effects. Clin. Rheumatol..

[44] Ikeda Y., Biro S., Kamogawa Y., Yoshifuku S., Eto H., Orihara K., Yu B., Kihara T., Miyata M., Hamasaki S. (2005). Repeated sauna therapy increases arterial endothelial nitric oxide synthase expression and nitric oxide pro-duction in cardiomyopathic hamsters. Circ. J..

[45] Park J.H., Lee S., Cho D.H., Park Y.M., Kang D.H., Jo I. (2013). Far-infrared radiation acutely increases nitric oxide production by increasing Ca(2+) mobilization and Ca(2+)/calmodulin-dependent protein kinase II-mediated phos-phorylation of endothelial nitric oxide synthase at serine 1179. Biochem. Biophys. Res. Commun..

[46] Anggard E. (1994). Nitric oxide: Mediator, murderer, and medicine. Lancet.

[47] Patrono C., FitzGerald G.A. (1997). Isoprostanes: Potential markers of oxidant stress in atherothrombotic disease. Arterioscler. Thromb. Vasc. Biol..

[48] Malek A.M., Izumo S., Alper S.L. (1999). Modulation by pathophysiological stimuli of the shear stress-induced up-regulation of endothelial nitric oxide synthase expression in endothelial cells. Neurosurgery.

[49] Aicher A., Heeschen C., Mildner-Rihm C., Urbich C., Ihling C., Technau-Ihling K., Zeiher A.M., Dimmeler S. (2003). Essential role of endothelial nitric oxide synthase for mobilization of stem and progenitor cells. Nat. Med..

[50] Kuehbacher A., Urbich C., Zeiher A.M., Dimmeler S. (2007). Role of Dicer and Drosha for endothelial microRNA expression and angiogenesis. Circ. Res..

[51] Weber M., Baker M.B., Moore J.P., Searles C.D. (2010). MiR-21 is induced in endothelial cells by shear stress and mod-ulates apoptosis and eNOS activity. Biochem. Biophys. Res. Commun..

[52] Ni C.W., Qiu H., Jo H. (2011). MicroRNA-663 upregulated by oscillatory shear stress plays a role in inflammatory re-sponse of endothelial cells. Am. J. Physiol. Heart Circ. Physiol..

[53] Di Stefano V., Zaccagnini G., Capogrossi M.C., Martelli F. (2011). microRNAs as peripheral blood biomarkers of cardiovascular disease. Vascul. Pharmacol..

[54] Li C., Pei F., Zhu X., Duan D.D., Zeng C. (2012). Circulating microRNAs as novel and sensitive biomarkers of acute myocardial Infarction. Clin. Biochem..

[55] Qu K., Wang Z., Lin X.L., Zhang K., He X.L., Zhang H. (2015). MicroRNAs: Key regulators of endothelial progenitor cell functions. Clin. Chim. Acta.

[56] Kaczmarek I., Deutsch M.A., Kauke T., Beiras-Fernandez A., Schmoeckel M., Vicol C., Sodian R., Reichart B., Spannagl M., Ueberfuhr P. (2008). Donor-specific HLA alloantibodies: Long-term impact on cardiac allograft vasculopathy and mortality after heart transplant. Exp. Clin. Transplant..

[57] Weiss M.J., Madsen J.C., Rosengard B.R., Allan J.S. (2008). Mechanisms of chronic rejection in cardiothoracic trans-plantation. Front. Biosci..

[58] Benatti R.D., Taylor D.O. (2014). Evolving concepts and treatment strategies for cardiac allograft vasculopathy. Curr. Treat. Options Cardiovasc. Med..

[59] Skoric B., Cikes M., Ljubas Macek J., Baricevic Z., Skorak I., Gasparovic H., Biocina B., Milicic D. (2014). Cardiac al-lograft vasculopathy: Diagnosis, therapy, and prognosis. Croat. Med. J..

[60] Borthwick L.A., Parker S.M., Brougham K.A., Johnson G.E., Gorowiec M.R., Ward C., Lordan J.L., Corris P.A., Kirby J.A., Fisher A.J. (2009). Epithelial to mesenchymal transition (EMT) and airway remodelling after human lung transplantation. Thorax.

[61] Chen P.Y., Qin L., Barnes C., Charisse K., Yi T., Zhang X., Ali R., Medina P.P., Yu J., Slack F.J. (2012). FGF reg-ulates TGF-beta signaling and endothelial-to-mesenchymal transition via control of let-7 miRNA expression. Cell Rep..

[62] Piera-Velazquez S., Jimenez S.A. (2012). Molecular mechanisms of endothelial to mesenchymal cell transition (En-doMT) in experimentally induced fibrotic diseases. Fibrogenes. Tissue Repair.

[63] D’Alessandro D.A., Kajstura J., Hosoda T., Gatti A., Bello R., Mosna F., Bardelli S., Zheng H., D’Amario D., Padin-Iruegas M.E. (2009). Progenitor cells from the explanted heart generate immunocompatible myocardium within the transplanted donor heart. Circ. Res..

[64] Hillebrands J.L., Klatter F.A., Rozing J. (2003). Origin of vascular smooth muscle cells and the role of circulating stem cells in transplant arteriosclerosis. Arterioscler. Thromb. Vasc. Biol..

[65] Sathya C.J., Sheshgiri R., Prodger J., Tumiati L., Delgado D., Ross H.J., Rao V. (2010). Correlation between circulating endothelial progenitor cell function and allograft rejection in heart transplant patients. Transpl. Int..

[66] Simper D., Wang S., Deb A., Holmes D., McGregor C., Frantz R., Kushwaha S.S., Caplice N.M. (2003). Endothelial progenitor cells are decreased in blood of cardiac allograft patients with vasculopathy and endothelial cells of non-cardiac origin are enriched in transplant atherosclerosis. Circulation.

